# Metagenomic analysis of gut microbiota in non-treated plaque psoriasis patients stratified by disease severity: development of a new Psoriasis-Microbiome Index

**DOI:** 10.1038/s41598-020-69537-3

**Published:** 2020-07-29

**Authors:** Ignacio Dei-Cas, Florencia Giliberto, Leonela Luce, Hernán Dopazo, Alberto Penas-Steinhardt

**Affiliations:** 1 Hospital Interzonal General de Agudos Presidente Perón, Servicio de Dermatología, Avellaneda, Argentina; 2 Psoriasis BsAs, Buenos Aires, Argentina; 3 Facultad de Medicina, Universidad de Buenos Aires, Buenos Aires, Argentina; 4 Facultad de Farmacia y Bioquímica, Departamento de Microbiología, Inmunología, Biotecnología y Genética, Cátedra de Genética, Laboratorio de Distrofinopatías, Universidad de Buenos Aires, Buenos Aires, Argentina; 5 Instituto de Inmunología, Genética y Metabolismo (INIGEM), CONICET - Universidad de Buenos Aires, Buenos Aires, Argentina; 6 CONICET, Biocódices, Buenos Aires, Argentina; 7 Laboratorio de Genómica Computacional, Departamento de Ciencias Básicas, Universidad Nacional de Luján, Luján, Argentina; 8 Instituto Universitario de Ciencias de la Salud Fundación H A Barceló, Buenos Aires, Argentina

**Keywords:** Medical research, Next-generation sequencing

## Abstract

Psoriasis is an immune-mediated skin disorder. Imbalance of gut microbial populations has been implicated in many diseases. We aimed to investigate whether there were differences in gut microbiota in psoriasis patients vs non-psoriasis controls and between psoriasis severity groups. 55 psoriasis patients and 27 controls were included. V3–V4 regions of the 16S rRNA gene of fecal samples were analyzed using Illumina MiSeq. Bioinformatic analysis was performed. We found changes in gut microbiome composition depending on their psoriasis status as determined by weighted unifrac (p < 0.05), in particular an increase in Firmicutes and depletion of Bacteroidetes in psoriasis patients. Additionally, the *Faecalibacterium* and *Blautia* genus were higher in psoriasis patients while *Bacteroides* and *Paraprevotella* in non-psoriasis controls (p < 0.05, LDA score > 2). Moderate-to-severe psoriasis patients had lower biodiversity than mild psoriatic patients (p = 0.049). No differences for beta-diversity were found. We developed a Psoriasis-Microbiota Index (PMI), which discriminated among psoriasis patients and controls with sensitivity: 0.78 and specificity: 0.79. Furthermore, we performed a meta-analysis with published data to validate this index. We demonstrated gut dysbiosis in psoriasis patients, suggesting a role in psoriasis pathophysiology. Furthermore, we developed a PMI with the potential to discriminate between psoriasis patients and controls across different populations, which could be used as a biomarker in the clinical practice.

## Introduction

Psoriasis is a chronic, immune-mediated inflammatory skin disease. It ranges in severity from a few scattered red, scaly plaques to involvement of almost the entire body surface^1^. Psoriasis is estimated to affect about 2–4% of the population in western countries, causes considerable psychosocial disability and has a major impact on patients’ quality of life^2,3^. Skin lesions are characterized by angiogenesis, an inflammatory reaction with recruitment of T cells into the skin, hyperproliferation of keratinocytes and altered epidermal differentiation^4^. Genetic and environmental factors are implicated in psoriasis, although, the exact etiology of the disease is not fully understood^5,6^.

The Human Microbiome Project (HMP) was initiated to fill a gap between our current understanding derived from Human Genome Project and actual physiological phenomenon. The HMP created a new view of ourselves as ‘super-organisms’ consisting of a human host and thousands of microbial symbionts^7^.

Imbalance of gut microbial populations or dysbiosis has important functional consequences and has been implicated in many digestive diseases, diabetes, obesity, metabolic syndrome, psoriatic arthritis, celiac disease, psychiatric disorders and others^8–13^.

There is a well-known relationship between psoriasis and other inflammatory diseases (obesity, inflammatory bowel disease, psoriatic arthritis, etc.)^14^. More importantly, bowel mucosa of active psoriasis patients without bowel symptoms show microscopic lesions, even when mucosa appeared macroscopically normal, with immune cellular infiltrates capable of producing pro-inflammatory cytokines^15^. Bacterial DNA translocation from the intestinal lumen has been described in patients with psoriasis suggesting that the gut microbiome may potentially act in skin diseases^16,17,18^.

Recent investigations point to the IL-23/Th17 axis as playing a major role in psoriasis pathogenesis^19^. The adhesion of specific members of gut microbiome to intestinal epithelial cells is found to be essential for the induction of Th17 cells^20–22^. Mice exposed to antibiotics showed inhibition of psoriasis induction by a dysregulation of gut and skin microbiota^23–25^.

There have been only limited studies of microbiota in psoriasis patients using molecular methods, which showed contradicting results regarding the most abundant taxa in the disease. These studies involved relatively small numbers of subjects, skin and gut microbiota and unmatched study designs^6,17,26–38^. Furthermore, none of existing reports evaluated changes in the gut microbiota among disease severity groups.

In the present study, we aimed to investigate whether the microbiota composition of non-treated chronic plaque psoriasis patients, as a group and divided according to disease severity, differs from non-psoriasis controls. We used strict inclusion and exclusion criteria. We included only patients with chronic plaque psoriasis and excluded patients with PsA and IBD (psoriasis comorbidities that are related to changes in the gut microbiota) and those patients under active systemic treatment, since there is evidence that methotrexate and biologic drugs induce compositional changes in the gut microbiota^39–41^. In addition, controls should not have family history of psoriasis in first degree relatives as genetics could also shape the gut microbiota^42^. Granted that there is abundant evidence that overweight or obese subjects have changes in their gut microbiota in relation to controls and that obesity and metabolic syndrome are comorbidities of psoriasis, we matched patients by sex, age and BMI^43,44^. Furthermore, we designed a Psoriasis-Microbiota Index (PMI) to discriminate patients against controls and performed a meta-analysis with previously published data to validate this index.

## Results

### Background of study cohort

This study included 55 untreated chronic plaque psoriasis patients and 27 unrelated non-psoriasis controls. The background of patients and controls are shown in Table 1. The patient group included 28 with mild disease and 27 with moderate-to-severe psoriasis. Table 2 represents the demographic data between mild and moderate-to-severe psoriasis groups, in which patients were comparable except for disease duration (longer in moderate-to-severe patients) and time since last relapse (longer for mild psoriasis).

**Table 1:** Characteristics of the sample.

	Psoriasis patients	Non-psoriasis controls	p
	n: 55	n: 27	
Age (years), mean ± SD	44.8 (16.9)	48.7 (18.8)	NS
Female (%)	49.1	57.7	NS
Male (%)	50.9	42.3	NS
Age of Psoriasis symptom onset (years), mean ± SD	30.5 (17.5)	NA	
Type 1 Psoriasis (%)	69.1	NA	
Last outbreak of Psoriasis symptoms (months), mean ± SD	4.2 (2.0)	NA	
Duration of Psoriasis (years), mean ± SD	14.3 (12.0)	NA	
Moderate-to-severe Psoriasis (%)	49.1	NA	
Hypertension (%)	29.1	NA	
Diabetes (%)	16.4	NA	
Weight, mean ± SD	81.8 (19.9)	75 (15.1)	NS
Heigh, mean ± SD	1.66 (0.1)	1.63 (0.1)	NS
BMI, mean ± SD	29.6 (5.5)	28.1 (5.2)	NS
Metabolic syndrome (%)	21.8	NA	
Overweight (%)	29.1	42.3	NS
Obesity (%)	45.5	30.7	NS
PASI, mean ± SD	9.9 (7.2)	NA	
BSA, mean ± SD	14.5 (18.5)	NA

**Table 2:** Demographic data in mild and moderate-to-severe psoriasis patients.

	Mild psoriasis patients	Moderate-to-severe psoriasis patients	p
	n: 28	n: 27	
Age, mean ± SD	41 ± 14.2	48.6 ± 18.9	NS
Female n: 27 (%)	42.9	55.6	NS
Male n: 28 (%)	57.1	44.4	NS
Age of Psoriasis symptom onset (years), mean ± SD	31 ± 15	29.9 ± 19.8	NS
Type 1 Psoriasis n: 38 (%)	47.4	52.6	NS
Last outbreak of Psoriasis symptoms (months), mean ± SD	4.7 ± 2.1	3.6 ± 1.7	0.04
Years with Psoriasis, mean ± SD	9.9 ± 8.7	18.6 ± 13.4	0.008
Hypertension n: 16 (%)	28.6	29.6	NS
Diabetes n: 9 (%)	17.9	14.9	NS
Weight, mean ± SD	85 ± 19.5	79.1 ± 20.2	NS
Heigh, mean ± SD	1.68 ± 0.1	1.64 ± 0.1	NS
BMI, mean ± SD	29.9 ± 5.8	29.5 ± 5.3	NS
Metabolic syndrome n: 12 (%)	21.4	22.2	NS
Overweight n: 16 (%)	28.6	29.6	NS
Obesity n: 25 (%)	46.4	44.4	NS
PASI, mean ± SD	3.7 ± 1.1	16.3 ± 4.8	0.000001
BSA, mean ± SD	2 ± 1.2	27.5 ± 19.2	0.000001

### Psoriasis vs non-psoriasis controls

#### Sequence analysis and comparison of microbial communities

**Figure 1:**
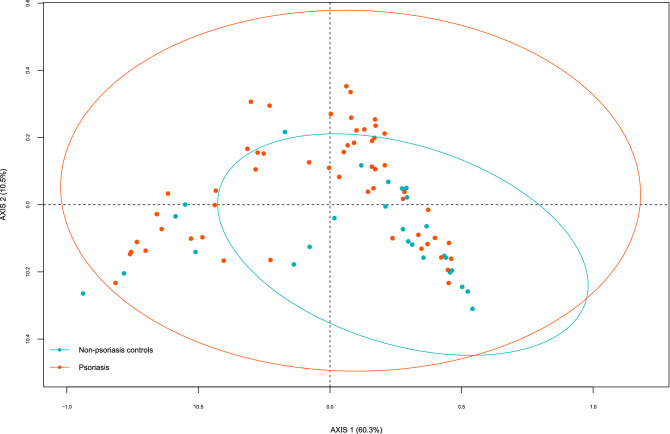
PCoA of beta-diversity values (Weighted Unifrac distances). Comparison of the gut microbiota from psoriasis patients and non-psoriasis controls. Ellipses show 95% confidence intervals.

The hypervariable region V3-V4 of bacterial 16S gene was sequenced using MiSeq-Illumina system, obtaining 152,939.46 ± 18,320.34 sequences per sample. Rarefaction plots reached an asymptotic state, indicating that the sequence depth was sufficient to represent the bacterial community richness and diversity (data not shown). Therefore, when we compared species richness (Chao1 index), there were no significant differences between psoriasis patients and controls. For beta-diversity as determined by Unifrac, we found significant differences between both groups, p = 0.034 for weighted UniFrac (Fig. 1) but not for unweighted UniFrac p = 0.255 (ADONIS).

**Figure 2:**
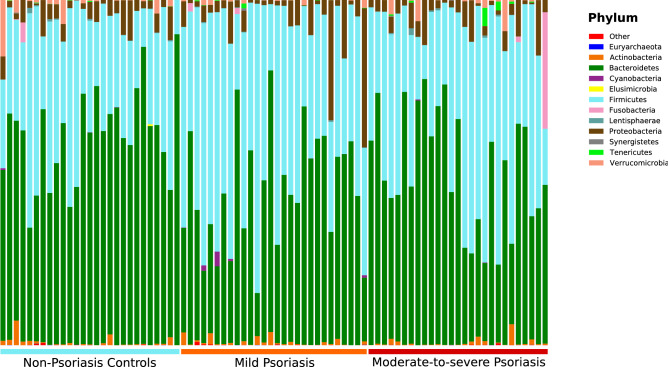
Bar plot showing the relative abundance of phyla distribution of each operational taxonomic unit (OTU) within samples.

Psoriasis patients differ from controls in the observed community structure. The dominant phyla in psoriasis patients were Bacteroidetes 47.1%, Firmicutes 44.6%, Proteobacteria 5.4%, Actinobacteria 0.8% and Fusobacteria 0.7%, while the principal phyla found in controls were Bacteroidetes 59.9%, Firmicutes 33.0%, Proteobacteria 4.2%, Verrucomicrobia 1.4% and Actinobacteria 0.8% (Fig. 2).

**Figure 3:**
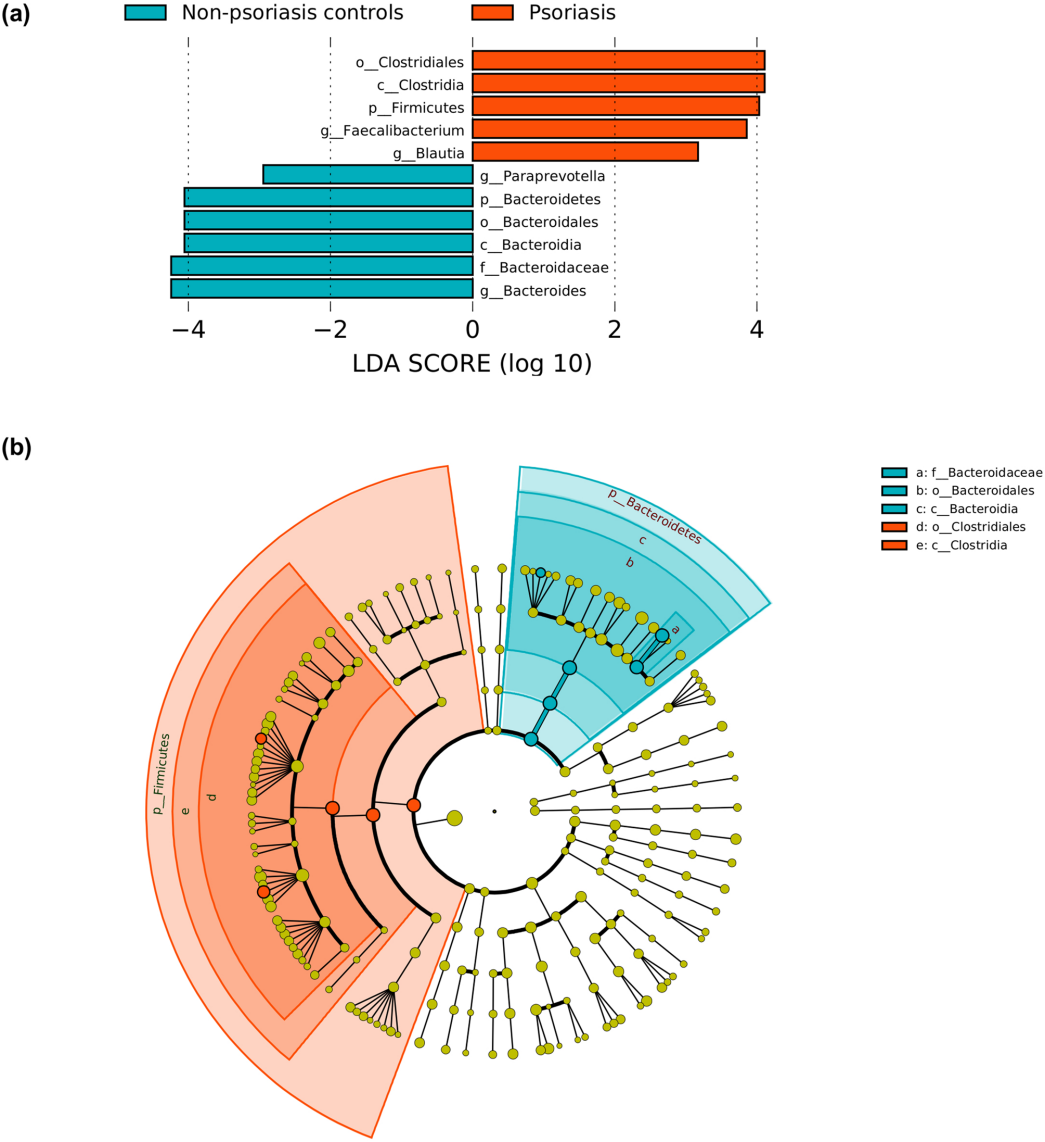
Plot from LEfSe analysis indicating enriched bacterial genus associated either with psoriasis patients (red) or non-psoriasis controls (blue). The length of the bar column represents the LDA score (**a**). Cladogram plotted from LEfSe analysis showing the differences in relative abundance of taxa at five levels between psoriasis patients vs non-psoriasis controls. (P < .05; LDA score 2) (**b**).

Phyla-level differences were detected between the two groups (control vs Psoriasis patients) including differences in Bacteroidetes and Firmicutes, with a Firmicutes to Bacteroidetes ratio of 0.63 ± 0.32 in non-psoriasis controls and 1.29 ± 0.81 in psoriasis patients (p = 0.0002). LefSe analysis revealed that these differences were mainly driven by changes in the *Bacteroides* and *Paraprevotella* genus which were more abundant in non-psoriasis controls while *Faecalibacterium* and *Blautia* in psoriasis patients (logarithmic LDA scores threshold was 2.0) (Fig. 3). We did not observe significant differences in gut microbiota associated with changes in age, weight and BMI.

### Mild vs moderate-to-severe psoriasis

Species richness in moderate-to-severe psoriasis patients was lower comparing with mild psoriasis patients (p = 0.049). Comparing the principal phyla detected, we did not find differences between both psoriasis groups (Supplementary Fig. S1).

We did not find differences in beta-diversity in mild vs moderate-to-severe psoriasis patients Supplementary Fig. S2. We did not observe significant differences for age, gender, age at psoriasis onset, years with psoriasis, hypertension, diabetes, weight, BMI, PASI and BSA. Only significant differences were found for metabolic syndrome (p = 0.002) in unweighted analysis.

### Psoriasis-Microbial index

**Figure 4:**
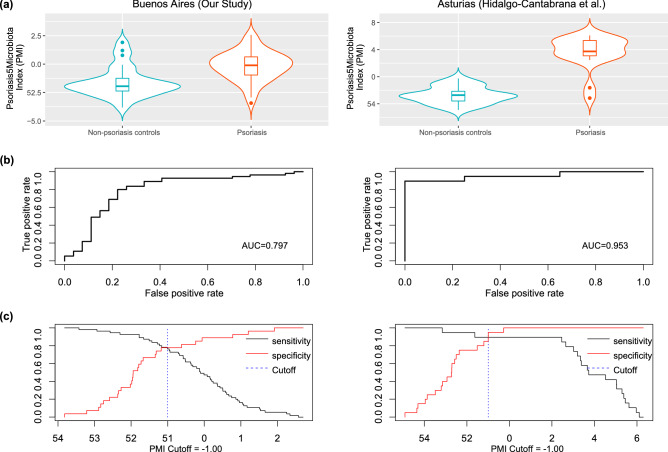
PMI distinguishes non psoriasis controls from psoriasis patients. Violin plot showing PMI in control and psoriasis fecal samples (**a**). Performance of cross-city prediction using each city-specific AD diagnosis model, as assessed via the area under the ROC curve (AUROC). The ROC curve of tenfold cross-validation was marked as blue lines and the ROC curve of the prediction as red lines. Performance of PMI, assessed via the area under the ROC curve (AUROC) (**b**). Sensitivity and specificity vs. PMI (Cutoff) plot in both populations (**c**).

Considering the results of relative abundance of different taxa, we generated the PMI to discriminate between psoriasis and non-psoriasis controls (Fig. 4a). We evaluated its applicability by ROC analysis. The Area Under the Curve (AUC) for the classification of Psoriasis (training dataset) was 0.797 (Fig. 4b), determining an optimal cut-off value of PMI = − 1.00 (sensitivity = 0.78 and specificity = 0.79) (Fig. 4c). When we applied the PMI according to psoriasis severity, the AUC was 0.849 and 0.743 for mild and moderate-to-severe psoriasis respectively.

### Meta-analysis

We validated this PMI using datasets from previously reported literature on PubMed. We identified 7 related 16S datasets^6,17,26,27,29,34,35^. Only the study of Hidalgo-Cantabrana et al*.*^34^ fulfilled the inclusion criteria.

When we applied the PMI to the downloaded sequence data from Hidalgo-Cantabrana et al.(test dataset)^34^ (Fig. 4a), the AUC was 0.953, Sensitivity = 0.89 and Specificity = 0.90, using cut-off value obtained with our dataset (PMI = − 1.00);(Fig. 4b). Sensitivity vs Specificity curves of both datasets were plotted in Fig. 4c showing concordant results, indicating that PMI would be a powerful tool capable of discriminating between patients with psoriasis and controls from different populations.

## Discussion

Intestinal dysbiosis is a possible actor in chronic inflammation, even in distant tissue sites, such as the skin. Imbalance in gut microbiota induces epithelial changes resulting in increased intestinal inflammation and altered gut permeability, which in susceptible individuals may trigger the development of different chronic disease states such as IBD, obesity, diabetes, multiple sclerosis, atopic dermatitis and cancer, among others^45–50^. However, until now, only a few studies have addressed this question in psoriasis^6,17,26,27,29,34–36,33^.

Our work demonstrates that there are differences in gut microbiota between psoriasis patients and non-psoriasis controls. We evaluated 55 untreated chronic plaque psoriasis patients (27 with moderate-to-severe psoriasis and 28 with mild disease), being according to our knowledge the study with the highest number of psoriasis patients and the first which evaluates changes in gut microbiota according to psoriasis severity based on well-defined strict criteria. We made a comparison of our study design with all the available publications on gut microbiota and psoriasis up to March 31, 2020 (Table 3).

**Table 3:** Study design of the available publications included in the meta-analysis (up to March 31, 2020).

	Our study	Codoñer et al.^20^	Tan et al.^29^	Hidalgo-Cantabrana et al.^37^	Chen et al.^30^	Huang et al.^6^	Scher et al.^32^	Shapiro et al.^38^
Publication year		2018	2018	2019	2018	2018	2015	2019
Population	Caucasian/Argentine	Caucasian/Spain	Asian/China	Caucasian/Spain	Asian/China	Asian/China	Caucasian/US	Caucasian/Israel
Psoriasis patients (n)	55	52	14	19	35	32	15 Ps / 16 PsA	24
Non-Psoriasis controls (n)	27	300 (from HMP)	14	20	27	64	17	22
Plaque psoriasis exclusive	yes	yes	yes	yes	yes	no	NA	NA
Matchead by	Age, sex & BMI	No	No	Age	Age, sex & BMI	No	Age & sex	Age, sex & comorbidities
Active systemic treatment	No	No	NA	No	Yes	Yes	No	Yes
Stratified by severity	Yes	No	No	No	NA	Yes	No	No
Concomitant PsA	no	NA	NA	NA	yes	no	yes	NA
16S region analyzed	V3–V4	V3–V4	V4	V2–V3	V3–V4	V4–V5	V1–V2	V4
Platform	Illumina	Illumina	Illumina	Ion Chef	Illumina	Illumina	Illumina	Illumina
Average reads	152,939	~ 85,000	~ 30,000	233,113	~ 85,000	NA	NA	~ 50,000
Raw data available at	PRJNA574485	Avaiable upon request to the corresponding author	NA	PRJNA517056	PRJNA379878	NA	NA	NA

Alpha-diversity has been observed to be decreased in a dysbiotic gut^51^. A lower microbial diversity has been found in some psoriasis studies^6,29,34^ but not by other investigators^17,26,27,35^. Our study does not show a lower alpha-diversity.

We found that Bacteroidetes and Firmicutes were the most prevalent phyla in patients and controls. However, there were significant differences between both phyla in psoriasis patients. The Firmicutes to Bacteroidetes ratio was 1.29 ± 0.81 in psoriasis patients and 0.63 ± 0.32 in non-psoriasis controls. In line with our results, other investigations showed a high Firmicutes:Bacteroidetes ratio^6,27,29,34,35^.

Short Chain Fatty Acid (SCFA) like acetate, propionate and butyrate, are known to regulate not only gut specific but also distant inflammatory responses through the induction of immune cells^52^. An increase in Firmicutes:Bacteroidetes ratio has been implicated in a higher acetate and lower butyrate production. Butyrate is the preferred fuel for the colonic epithelial cells and the major regulator of cell proliferation and differentiation, and has important anti-inflammatory, antioxidant and anti-carcinogenic functions^53^. Low levels of butyrate may affect the integrity of the mucous layer compromising the gut epithelial barrier and enhance chronic colonic and systemic inflammation^54^.

Beta-diversity showed that genus *Faecalibacterium* and *Blautia* (both belong to the phylum Firmicutes, class Clostridia and order Clostridiales) were the most relevant genus in psoriasis patients that discriminated against non-psoriasis controls. *Faecalibacterium prausnitzii* (*F. prausnitzii*) can regulate T helper 17 cell (Th17)/regulatory T cell (Treg) differentiation and has been consistently reported as one of the main butyrate producers found in the intestine^53,55^. The role of *F. prausnitzii* in maintaining immune and physiological functions promoted this bacterium as a next generation probiotic^56^.

In psoriasis, a decrease in relative abundance of *F. Prausnitzii* has been reported in some studies^30,34^, but not by other investigators^17,27,35^. In our study the genus *Faecalibacterium* showed higher values in psoriasis patients. Lopez-Siles et al. determined that *F. prausnitzii* includes two phylogroups and recent studies suggest that other *Faecalibacterium* genus and species could not be ruled out^53,57,58^. The relative abundance, as well as which phylum and species of *Faecalibacterium* population are disbalanced in different diseases, makes it difficult to establish the use of a single bacteria as a general biomarker for all diseases. The use of *F. prausnitzii* as a gold standard of a healthy gut microbiota is limited^53^.

The genus *Blautia* includes obligate anaerobic intestinal commensal bacteria that belong to the family Lachnospiraceae and includes more than 100 different species^59,60^. *Blautia* are important members of the healthy human gut microbiota^61^. Jenq et al. found a lower mortality due to a graft versus-host disease after allogeneic blood/marrow transplantation among patients with high abundance of *Blautia* and Bajaj et al.found that *Blautia* was one of the bacteria associated with improved outcomes in patients with liver cirrhosis^62,63^. Genus *Blautia* has been also related to cancer. Chen et al.reported a detrimental association between lower concentrations of *Blautia* in the gut and colorectal cancer^64^. On the contrary, Luu et al. found that higher levels of *Blautia* were associated with poor prognosis in patients with early-stage breast cancer^65^. Considering the limited data available on *Blautia* and the huge number of species reported, we can not explain the reasons why genus *Blautia* was increased in our work. Additional data are required to determine their true role in human diseases.

The fact that most relevant genus in psoriasis patients that discriminated against non-psoriasis controls were *Faecalibacterium* and *Blautia,* taxa producing high levels of butyrate, contradicts the traditional association of butyrate producers observed in diseases such as IBD^66,67^. Therefore, results highlight the need for additional research given the observational nature and limits of 16S used in this study.

In our control group, the predominant genus were *Bacteroides* and *Paraprevotella*. These bacteria differ only in family (Bacteroideaceae for *Bacteroides* and Prevotellaceae for *Paraprevotella*)^68,69^. Increasing evidence proposes that *Bacteroides* harness complex recalcitrant glycans^70^. SCFAs are the major metabolic products of anaerobic fermentation of glycans by gut bacteria and have been shown to impact on the host physiology^71^. The beneficial effect of *Bacteroides* is consistent with our findings, where this genus was increased in controls and depleted in psoriasis patients.

There is evidence that age, diet, geographical location, genetics and antibiotics, among other factors, influence gut microbiota^72,73^. We selected unrelated controls matched by sex, age and BMI to moderate-to-severe psoriasis patients, living in the same area and with a similar diet in order to reduce those confounding factors. We did not find differences in beta-diversity according to personal features, so we postulate that changes in gut microbiota would then be dependent on psoriasis and not on other covariates.

We found that patients with moderate-to-severe psoriasis had a lower diversity (species richness) than patients with mild disease, although this difference was subtle. Only Huang et al.also studied whether the composition of the intestinal microbiota differed depending on the severity of the disease and they found that the genus *Bacteroides* was increased in patients with psoriasis and that it was characteristic of the subgroup with severe disease^6^. In our study, the genus *Bacteroides* was found to be diminished in patients with psoriasis but no differences were found between mild and moderate-to-severe psoriasis patients. For example, these distinctions could be due to different inclusion criteria.

When we compared whether the microbiota of patients with mild psoriasis vs patients with moderate-to-severe psoriasis was affected by age, sex, age at onset of the disease, years of illness and comorbidities such as hypertension or diabetes, we could not establish differences between both severity groups. These results could also explain that changes of the gut microbiota in psoriasis would be dependent on the presence of the disease and would not be affected by its severity.

Codoñer et al., Shapiro et al.and Hidalgo-Cantabrana et al.reported similar results to our study regarding the bacteria genus increased in psoriasis and controls^17,34,35^. This concordance suggests that there is probably a core gut microbiota in psoriasis patients. Unfortunately, not all the studies met the inclusion criteria for the meta-analysis. Codoñer et al. did not use a control group from the same geographic location as they used publicly available data from The Human Microbiome Project and the raw data from Shapiro et al.were not available. This serves as another example of the importance of unrestricted access to raw sequencing data, which has been already recognized by the scientific community^74^. Despite variations among Hidalgo-Cantabrana et al.and our study, a psoriasis model can be applied across populations from different geographical locations. The proposed PMI proved to be able to discriminate between psoriasis and controls across cities and continents with an optimal cut-off value of PMI = − 1.00.

Given that general dermatologists are able to make a diagnosis of psoriasis with a simple physical exam, the diagnostic applicability of the test will have to await further clinical experience. The PMI represents, a step forward as a combined practical, ready to use, clinical and research tool. The index will allow us to gain more knowledge on the microbial component of psoriasis and provide the possibility of increasing our understanding of the role played by the microbiome in the disease process. Moreover, as PMI was only tested in 2 cohorts of non-treated patients, we cannot exclude its role as a biomarker for evaluating treatment response. Further studies of metagenome shotgun sequencing at the species/strain levels might be useful for the update and improvement of the developed PMI.

In summary, our findings demonstrate variations in gut microbiota profiles between non-treated plaque psoriasis patients and non-psoriasis controls. This results suggest that it is likely that altered gut microbiota plays a pathophysiological role in psoriasis. However, whether modulation of gut microbiota could modify the course of the disease remains to be explored. This study is unique in being the first to propose a PMI with the ability to discriminate between psoriasis patients and age-sex-and BMI matched controls and between samples from communities of different continents. Further studies are needed to better interpret the role of the PMI as a potential biomarker test in psoriasis, and to test this index in larger and diverse populations to confirm its validity.

## Methods

### Study participants

This cross-sectional study recruited unrelated individuals, including consecutive chronic plaque psoriasis patients and non-psoriasis controls. Controls were matched to moderate-to-severe psoriasis patients according to sex, age (± 2 years) and Body Mass Index (BMI; ± 1). Participants were caucasian, above 18 years old and from the same geographical location. Samples were collected between October 2017 and April 2018.

Psoriasis patients were subdivided based on their severity in mild and moderate-to-severe psoriasis. Mild psoriasis was defined as actual Body Surface Area covered by psoriasis (BSA) < 10%, Psoriasis Area and Severity Index (PASI) < 10, Investigator Global Assessment (IGA) < 3 and absence of episodes of moderate-to-severe psoriasis in the past. Moderate-to-severe psoriasis was defined as BSA ≥ 10%, PASI ≥ 10 and IGA ≥ 3.

Two visits were conducted over a period of 4 weeks to take a detailed assessment of psoriasis, medical history, and a complete physical exam, including PASI, IGA and BSA involvement. Type 1 psoriasis was defined if the symptoms began on or before age 40 years; a BMI ≥ 25 was considered as excessive weight and BMI ≥ 30 as obesity.

Key exclusion criteria for psoriasis patients included concomitant diagnosis of psoriatic arthritis according to CASPAR criteria, inflammatory bowel disease (IBD), current topical treatment, systemic treatment for psoriasis (including phototherapy) 3 months previous to sample collection, assuming that immunosuppression could modify gut microbiota.

The exclusion criteria for controls were the presence of other dermatosis, family history of psoriasis in first degree relatives, immunological disorders, hypertension, fatty liver disease, diabetes mellitus, malignancy, any other serious internal disease, smoking and alcohol abuse.

Exclusion criteria applied to all groups were: antibiotic therapy 3 months previous to sample collection, extreme diet, consumption of probiotics, positive HIV test or any gastrointestinal tract surgery leaving permanent residua.

### Sample collection and DNA extraction

All participants were apprised for the stool sampling collection method by receiving a standardized protocol for the collection of approximately 5 g of stool in a sterile bacteriostatic buffer tube^75^. Participants were asked to collect samples 24 h before the second visit. DNA extraction was performed from 200 mg of feces using QIAamp-PowerFecal DNA-Kit.

### Comparison of microbial communities and sequence analysis

Hypervariable regions V3–V4 of the 16S rRNA gene were amplified with primers 337F/805R and sequenced in paired-end mode using a MiSeq sequencer (Illumina^Ⓡ^), warranting an average of 152,939 sequences per sample.

De-multiplexed reads were quality trimmed using Trimmomatic(V0.36)^76^. Sequences generated were analyzed using Quantitative Insights Into Microbial Ecology (QIIME) version 1.9.1 software package^77^. For this purpose, the sequences obtained were compared with those from Greengenes 13_8 database^78^. Chimeric sequences were filtered using VSEARCH^79^. Operative Taxonomic Units (OTUs) were assigned to each read with an open_reference OTU picking process. SortMeRNA (v2.1)^80^ was used for the reference OTU picking steps (with sortmerna_coverage = 0.8) and sumaclust (v1.0.20)^81^ for the de novo OTU picking steps (with 10% of the failures subsampled). Low-confidence OTUs called by < 0.1% of the reads were removed using the script remove_low_confidence_otus.py^82^. An average of 29,872.93 ± 6,452.75 mapping high-quality sequences were obtained, leading to 455.91 ± 126.99 unique OTUs per sample. For multiple comparisons, p-values were adjusted by Bonferroni correction^83^. To compare microbial communities in different sample groups, we used Unifrac algorithm^84^. Differences on beta-diversity were assessed using ADONIS. In order to compare the relative abundance of the different taxa between groups, we performed Linear Discriminant Analysis (LDA) effect implemented in LEfSe^85^ .

### Psoriasis-Microbiome Index development

PMI was defined as the logarithm of total abundance of organisms increased in psoriasis over total abundance of organisms decreased in psoriasis for all samples (at genus level) using the compute_taxonomy_ratio.py script^86^. Then, we evaluated how these PMI performed for classification subjects by psoriasis status through Receiver Operating Characteristic (ROC) analysis (training dataset). ROC analysis was performed using ROCR package (RStudio version 1.1.453)^87^. Cut-off value was selected as the point where the sensitivity and specificity functions intersect each other, i.e. jointly maximizing the sensitivity and specificity of PMI.

### Meta-analysis

We performed a systematic literature search of PubMed databases up to March 31, 2020 using the following terms: “Psoriasis” and “gut microbiota” or “gut microbiome”. The study inclusion criteria were: Case–control studies with publicly available raw 16S data and metadata, indicating case/control status for each sample. Studies including patients with other clinical forms different from plaque psoriasis and patients under systemic treatment (DMARDS and biologics) were excluded.

### Data accession

Raw sequences of 16S rRNA gene reported in this article have been deposited in NCBI Short Read Archive (SRA) and are accessible under the accession number PRJNA574485.

### Ethical statement

This study received approval by the Ethics Committee of Hospital Español, Buenos Aires Argentina according to local regulations and Helsinki declaration. Written informed consent was obtained from all study participants.

## Supplementary information

Supplementary file1 (PDF 115 kb)
