# Supplementary material for: Metagenomic analysis of gut microbiota in non-treated plaque psoriasis patients stratified by disease severity: development of a new Psoriasis-Microbiome Index
Source: Sci Rep. 2020 Jul 29;10:12754. doi: 10.1038/s41598-020-69537-3 (PMC7391695; doi:10.1038/s41598-020-69537-3)
Supplement: Supplementary file 1 — Supplementary file1 (PDF 115 kb) [file 41598_2020_69537_MOESM1_ESM.pdf]

Metagenomic analysis of gut microbiota in non-treated plaque psoriasis patients stratified by disease severity. Development of a new Psoriasis-Microbiome Index.

Authors

Dei-Cas, Ignacio - ideicas@intramed.net[1]

Giliberto, Florencia - gilibertoflor@gmail.com[2 3]

Luce, Leonela - leonelaluce@gmail.com[2 3]

Dopazo, Hernán - hernan.dopazo@biocodices.com[4]

Penas-Steinhardt, Alberto – pufetin@gmail.com[5,65]

Author information

1. Hospital Interzonal General de Agudos Presidente Perón, Servicio de Dermatología; Psoriasis BsAs, Psoriasis; Universidad de Buenos Aires, Facultad de Medicina

2. Universidad de Buenos Aires. Facultad de Farmacia y Bioquímica. Departamento de Microbiología, Inmunología, Biotecnología y Genética, Cátedra de Genética, Laboratorio de Distrofinopatías. Buenos Aires, Argentina.

3. CONICET-Universidad de Buenos Aires. Instituto de Inmunología, Genética y Metabolismo (INIGEM). Buenos Aires, Argentina.

4. CONICET, Biocódices.

5. Laboratorio de Genómica Computacional, Departamento de Ciencias Básicas, Universidad Nacional de Luján, Argentina

6. Instituto Universitario de Ciencias de la Salud Fundación H A Barceló.

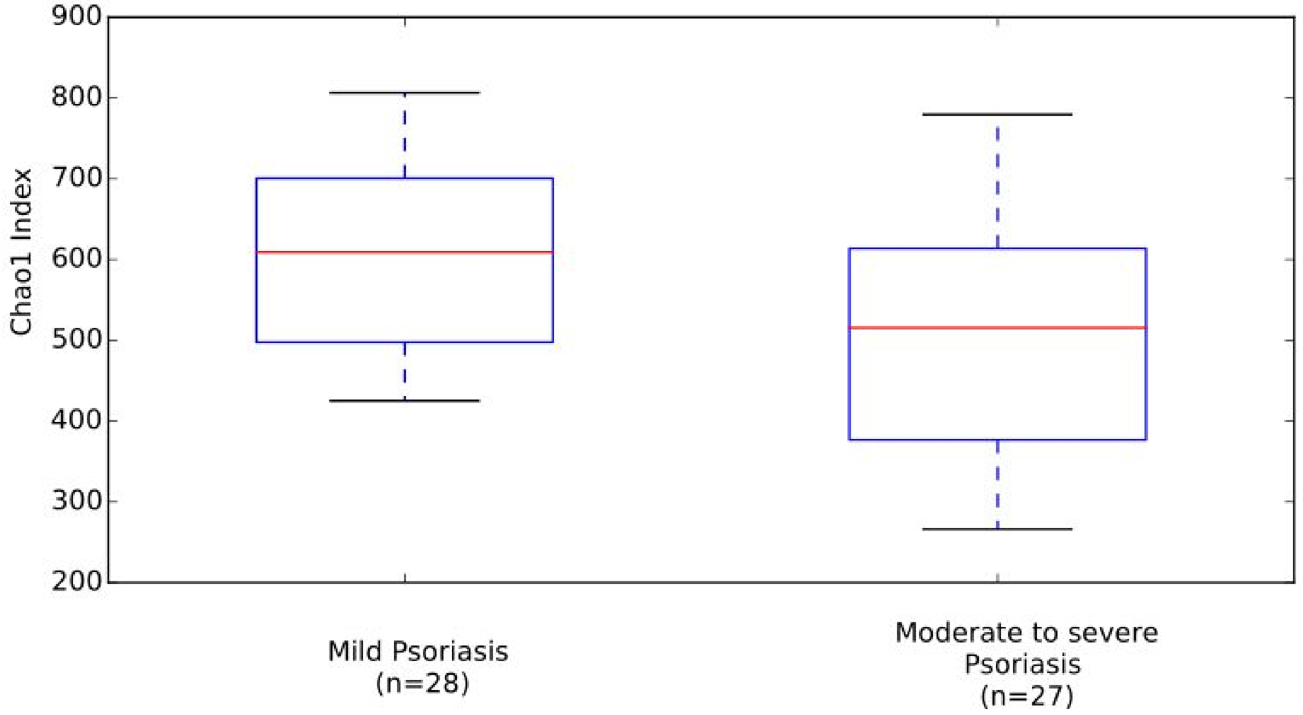

Supplementary Figure S1. Alpha diversity plot. Chao1 richness estimator between mild and moderate to severe Psoriasis

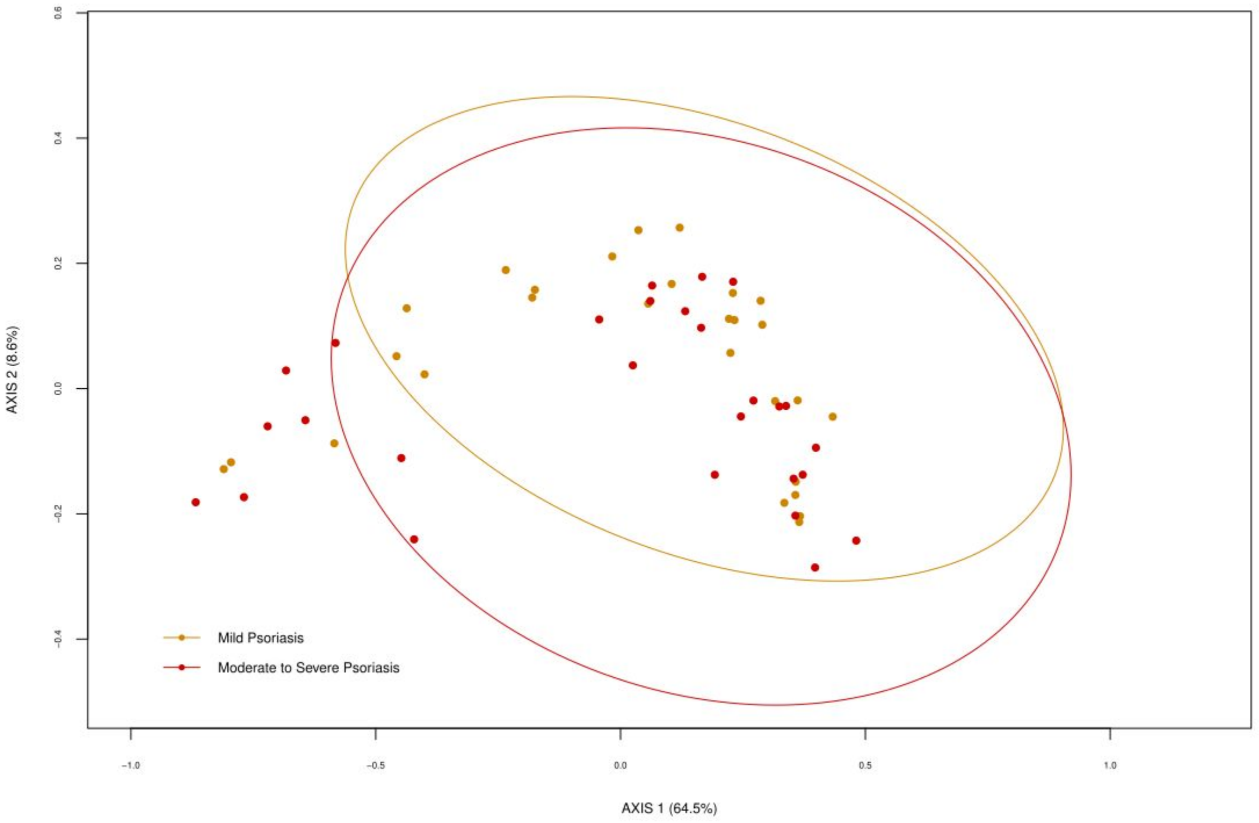

Supplementary Figure S2. PCoA of beta-diversity values (Weighted Unifrac distances). Comparison of the gut microbiota from mild psoriasis (orange) and moderate to severe psoriasis (red) patients. Ellipses show 95% confidence intervals.
